# Procedural success and safety of a standardized less invasive surfactant administration protocol using fentanyl premedication and video laryngoscopy in preterm infants <32 weeks’ gestation

**DOI:** 10.3389/fped.2025.1704163

**Published:** 2025-10-31

**Authors:** Mircea-Horia Popa-Todirenchi, Benedikt Bubl, Fabian Keller, Marta Busso, Sophie Jaisli, Lisa Marie Bünte, Marc-Alexander Oestreich, André Kidszun

**Affiliations:** ^1^ Division of Neonatology, Department of Paediatrics, Inselspital, Bern University Hospital, University of Bern, Bern, Switzerland; ^2^ Department of Paediatrics, Inselspital, Bern University Hospital, University of Bern, Bern, Switzerland; ^3^ University Children’s Hospital Basel (UKBB), Basel, Switzerland

**Keywords:** respiratory distress syndrome (RDS), noninvasive ventilation, analgesia, pulmonary surfactants, atropine, neonate, sedation, minimally invasive surfactant therapy (MIST)

## Abstract

**Background:**

Less Invasive Surfactant Administration (LISA) has been established for treating respiratory distress syndrome (RDS) in preterm infants, but the impact of pharmacological analgesia or video laryngoscopy on procedural success and safety remains unclear. We evaluated procedural success and adverse events of LISA performed according to a standardized protocol incorporating intravenous opioid analgesia and video laryngoscopy assistance.

**Methods:**

This single-center, retrospective, observational study was conducted at a tertiary neonatal unit from September 2021 to August 2023. All live-born infants <32 weeks’ gestation were screened. Exclusion criteria were parental objection to data use, missing primary outcome data, major congenital malformations, prenatal palliative care decisions, outborn transfered after 24 h of life or already receiving invasive ventilation. The LISA protocol comprised the administration of intravenous fentanyl (1 µg/kg) 3–5 min prior to video laryngoscopy, followed by delivery of Poractant alfa (100–200 mg/kg) via a thin oral catheter. The primary endpoint was first-attempt success. Secondary endpoints comprised the frequency of procedure-related adverse events, including bradycardia (HR <80 bpm), desaturation (SpO₂ < 80%), number of LISA attempts, arterial hypotension, and LISA failure, defined as intubation with subsequent mechanical ventilation within 72 h.

**Results:**

Of 243 live-born infants, 63 were excluded from analysis. Of the remaining 180 infants 84 (47%) received LISA, 45 (25%) were intubated in the delivery room and 51 (28%) remained on non-invasive ventilation without surfactant. In the LISA cohort (median gestational age: 29 + 3 weeks; mean birth weight: 1,176 g), fentanyl was administered to 83/84 patients (99%), and video laryngoscopy was used in 82/84 patients (98%). First-attempt success was achieved in 52/84 (62%) infants. Attending physicians had a significantly higher probability of first-attempt success than interns (OR 4.86, 95% CI 1.40–16.80, *p* = 0.013). Periprocedural bradycardia occurred in 6 infants (7%), desaturation in 65 infants (77%) and post-procedure hypotension in 6 infants (7%). LISA failure occurred in 14/84 (17%) infants.

**Conclusion:**

This study provides detailed data on the procedural quality of LISA when opioid analgesia and video laryngoscopy are used. This data may inform future trials investigating analgesia strategies, safety, and long-term outcomes.

## 1 Introduction

Respiratory Distress Syndrome (RDS), caused by surfactant deficiency, remains an important challenge in the management of preterm infants and is a major cause of morbidity and mortality (1). Surfactant replacement therapy is one cornerstone of treatment, historically administered via an endotracheal tube, often requiring mechanical ventilation (MV). The intervention is associated with risks of volutrauma and barotrauma, contributing to the development of bronchopulmonary dysplasia (BPD) (2).

In recent years, non-invasive strategies have been developed toward respiratory care of preterm infants. Less Invasive Surfactant Administration (LISA), also known as Minimally Invasive Surfactant Therapy (MIST), has emerged as a promising alternative (1, 3–7). This technique involves delivering surfactant through a thin catheter to spontaneously breathing infants maintained on non-invasive respiratory support, typically nasal continuous positive airway pressure (nCPAP) (1). Systematic reviews and large-scale network meta-analyses provide evidence that LISA is associated with a significant reduction in the composite outcome of death or BPD when compared with traditional intubation or nCPAP alone (1, 8). LISA is now recommended in international guidelines as the preferred method for surfactant delivery in appropriately selected preterm infants (1, 9).

Despite the established benefits of LISA, a significant controversy persists regarding the use of premedication (1, 6, 9). The procedure requires direct laryngoscopy to visualize the vocal cords for catheter placement, a procedure potentially painful and physiologically stressful for the infant (10, 11). Awake laryngoscopy can provoke adverse physiological responses, including hypertension, bradycardia, desaturation, and fluctuations in intracranial pressure (9, 11). Furthermore, the impact of such procedural pain is not transient. It has been shown that cumulative exposure to painful procedures during the neonatal period is associated with long-term adverse effects on brain architecture, including alterations in white matter maturation and regional brain volumes, which may contribute to subsequent neurodevelopmental impairment (10). Some consider adequate analgesia for painful medical procedures as standard of care (10, 12).

However, the administration of analgesic medication is not without risk. The main concern is the potential for respiratory depression, which could compromise the spontaneous respiratory drive that is important for the success of the LISA technique (1, 6, 11). Opioid analgesics have, however, been associated with a risk of smaller cortical and cerebellar volumes (13) as well as with poorer neurodevelopmental outcomes (14). Other potential side effects include hypotension and, with certain opioids like fentanyl, chest wall rigidity (15). This clinical context has led to wide international variation in practice, with a large group of providers opting against the routine use of premedication for LISA (1, 9).

This study aimed to evaluate first-attempt success rates and the incidence of procedure-related adverse events in preterm infants with a gestational age of less than 32 weeks undergoing LISA with a standardized protocol combining video laryngoscopy and routine premedication with fentanyl and atropine.

## 2 Methods

This single-center, retrospective observational study was conducted at a tertiary care academic neonatal unit over a two-year period from September 1, 2021, to August 31, 2023. The study was approved by the responsible cantonal ethics committee (2023-01965).

All live-born infants with a gestational age of less than 32 weeks during the study period were screened. Infants were excluded from analysis if there was parental objection of the use of data, if they had missing primary outcome data, major congenital malformations, prenatal palliative care decisions, outborn patients transferred after 24 h of life or outborn patients already on invasive ventilation. The respiratory management analysis classified eligible infants into three groups: those who received LISA, those intubated in the delivery room, and those who remained on non-invasive support without surfactant administration. The analysis focuses on the subgroup of infants who received LISA.

LISA was implemented in the unit on the 1st of September 2021 and performed according to a standardized departmental protocol with the indication for surfactant administration according to the infants' gestational age. Infants under 28 weeks of gestation received surfactant if they required a fraction of inspired oxygen (FiO_2_) > 0.21 to achieve an oxygen saturation (SpO_2_) ≥ 90% or if the Silverman score (16) exceeded 5. Preterm infants between 28 and <32 weeks of gestation were eligible for surfactant administration if they required an FiO_2_ ≥ 0.30 to achieve an SpO_2_ ≥ 90% or they had a Silverman score >5 combined with an FiO_2_ > 0.21. Infants were stabilized on nasal continuous positive airway pressure (nCPAP) prior to the procedure. Premedication consisted of intravenous fentanyl (1 µg/kg) and atropine (0.005 mg/kg) administered 3–5 min before laryngoscopy. The vocal cords were visualized using a video laryngoscope (InfantView® Accutronic Switzerland or C-MAC® Karl Storz SE & Co. KG Germany), and a thin catheter (LISAcath® Chiesi Farmaceutici S.p.A. Italy or Surfcath™ Vygon SA France) was inserted into the trachea. Poractant alfa (Curosurf®) was instilled at a dose of 100–200 mg/kg. Throughout the procedure, heart rate (HR), SpO₂, and blood pressure (BP) were continuously monitored.

The primary endpoint was first-attempt success, defined as passage of the catheter through the vocal cords followed by intra-tracheal instillation of surfactant on the first laryngoscopy attempt. Secondary endpoints included procedure-related outcomes: frequency of bradycardia (HR <80 bpm), frequency of desaturation (SpO₂ < 80%), number of LISA attempts, need for positive pressure ventilation (PPV) during the procedure, and arterial hypotension. Other secondary outcomes included procedure failure (need for intubation and mechanical ventilation within 72 h following LISA), early death (before 28 days of life), intraventricular hemorrhage (IVH) greater than grade II, pneumothorax, and duration of respiratory support and oxygen supplementation.

Data were collected retrospectively from electronic and physical medical records, including demographic information, baseline characteristics, and all primary and secondary outcome variables. Descriptive statistics were used to summarize patient characteristics and outcomes. Continuous variables were expressed as mean ± standard deviation (SD) or median and interquartile range (IQR), as appropriate. Categorical variables were presented as counts and percentages (n, %). Missing data were omitted from the analysis.

In order to potentially identify factors influencing the primary outcome we performed an univariate analysis for the association with the binary primary outcome for: sex, gestational age, birthweight, small for gestational age (SGA), lung maturation, dichotomized APGAR at 5 min (<7 or ≥7), preterm premature rupture of membranes (PPROM), arterial pH, time to LISA, and FiO_2_ before LISA. Based on a threshold of *p* < 0.25 for the initial screening we included FiO_2_ before LISA, and due to clinical relevance, gestational age as well as small for gestational age (SGA) in a penalized logistic regression (Firth logit) model. The lenient threshold was chosen to avoid excluding potentially important predictors that could have stronger correlations in a multivariable context. A *post-hoc* analysis was performed in order to identify potential impact of the procedure provider on the primary outcome. The differences between procedure providers were evaluated using Pearson's *χ*^2^ Test and multivariate logistic regression. Statistical analyses were performed using STATA 16 (StataCorp, College Station, USA). A *p*-value of <0.05 was considered statistically significant.

## 3 Results

**Figure 1:**
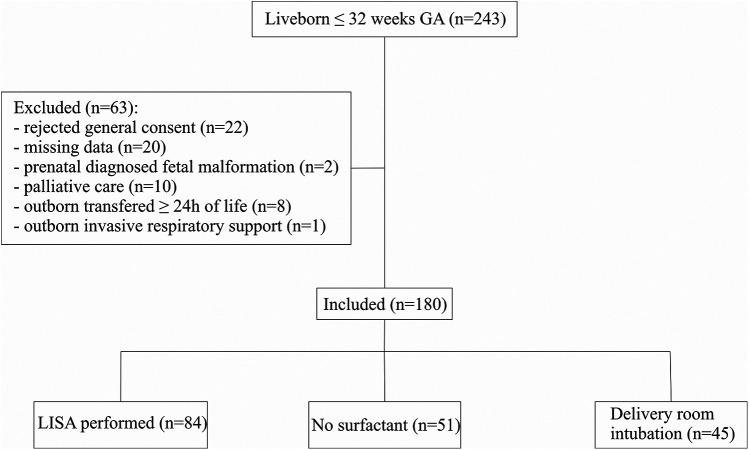
Flow chart.

During the two-year study period, 243 live-born infants were screened. After applying exclusion criteria, a total of 180 patients were included in the analysis. Of these, 84 infants received surfactant via the LISA, 45 were intubated in the delivery room, and 51 had no clinical indication for surfactant administration (10/51 infants never requiring CPAP) (Figure 1).

The baseline demographics and pre-intervention clinical characteristics of the 84 infants in the LISA group are described in Table 1.

**Table 1:** Demographic and clinical characteristics of the LISA cohort (*n* = 84).

Characteristic	Value
Demographics	
Gestational age (weeks), median, [min, max]	29 3/7 [24 0/7–31 6/7]
Birth weight (g), mean ± SD	1,176 ± 359
Male gender, *n* (%)	49 (58%)
Small for gestational age, *n* (%)	8 (10%)
Maternal & delivery data	
Antenatal steroids, *n* (%)	77 (92%)
Caesarean section, *n* (%)	78 (93%)
PPROM, *n* (%)	23 (27%)
Pre-LISA status	
Time to surfactant (min), median [min, max]	55 [25–960]
Silverman score, median [min, max]	7 [1–10]
FiO2​ before LISA, median [IQR]	0.30 [0.30–0.45]
nCPAP (cm H2O), median [min, max]	6 [5–9]
SpO2​ before LISA (%), mean ± SD	93.1 ± 3.0

IQR, interquartile range; nCPAP, nasal continuous positive airway pressure; PPROM, preterm premature rupture of membranes; SD, standard deviation.

The median gestational age was 29 3/7 weeks (IQR 27 2/7–30 5/7), and the mean birth weight was 1,176 g (SD ± 359 g). Prior to the procedure, the median Silverman score was 7 (IQR 6–8) and the mean FiO2 was 0.39.

Premedication with fentanyl and atropine was administered in 83 of 84 infants (99%), 1 infant receiving Esketamine as sedation because of important pre-procedure arterial hypotonia. Video laryngoscopy was used in 82 of 84 procedures (97%) with technical problems being mentioned as reason for use of standard laryngoscopy.

### 3.1 First-attempt success rate

During the procedure there was at least one attending physician as well as an intern or a fellow present. The attending could freely decide who would provide LISA, with the prerequisite that the provider had been previously trained on the mannequin. Of the 84 LISA procedures, 52 (62%) were successful at the first attempt. Attending physicians led 34/48 (71%) of their procedures to success, compared with 13/21 (62%) for neonatology fellows and 5/15 (33%) for pediatric interns. The mean number of attempts required for successful administration was 1.57 with a median of 1. Multivariate analysis revealed a significant influence of training level on the first-attempt success rate [LR *χ*^2^(2) = 6.75, *p* = 0.034, pseudo-*R*^2^ = 0.062]. Attendings had a significantly higher probability of first-attempt success than interns (OR 4.86, 95% CI 1.40–16.80, *p* = 0.013), while fellows showed a non-significant but similar trend in comparison to interns (OR 3.71, 95% CI 0.90–15.26, *p* = 0.069). The predicted probability of first-attempt success was 33% for interns, 65% for fellows, and 71% for attendings.

Univariate logistic regression analysis showed no significant association between LISA first-attempt success and the covariates gestational age, FiO_2_ before LISA, or small for gestational age (SGA). In the multivariable model, no predictor reached statistical significance (Table 2).

**Table 2:** Multivariable analysis of first-attempt success.

Predictor	OR (95% CI)	*p*-value
Gestational age	0.99 (0.96–1.02)	0.53
Higher FiO_2_ before LISA	0.17 (0.01–2.29)	0.18
SGA	0.49 (0.12–2.04)	0.32

SGA, small for gestational age.

### 3.2 Procedure-related adverse events

Desaturation (SpO₂ < 80%) occurred in 65 of 84 infants (77%). Bradycardia (heart rate <80 bpm) was observed in 6 of 84 infants (7%). Arterial hypotension following the procedure was documented in 6 of 84 infants (7%). In 20 of 84 infants (24%), brief positive pressure ventilation was applied, and in 49 of 84 infants (58%), FiO₂ was temporarily increased and/or tactile stimulation was provided. Chest wall rigidity was not a pre-specified adverse event, either in the standard clinical documentation, or in the study protocol. However, there is no mention of chest wall rigidity in the documented free comments on adverse events of the procedure.

### 3.3 Efficacy and morbidities

The procedural failure rate, defined as the need for intubation and mechanical ventilation within 72 h after LISA, was 17% (14/84). 83% of infants were managed without mechanical ventilation during the first three days of life. Rates of major morbidities are summarized in Table 3.

**Table 3:** LISA efficacy and Major morbidities (*n* = 84).

Outcome	Value
Efficacy outcome	
FiO_2_ 1 h after LISA, median [IQR]	0.21 [0.21–0.30]
FiO_2_ 6 h after LISA, median [IQR]	0.21 [0.21–0.21]
LISA Failure (MV within 72 h), *n* (%)	14 (17%)
Major morbidities & mortality	
Intraventricular hemorrhage (> Grade II)	7 (8%)
Pneumothorax, *n* (%)	2 (2%)
Early death (< 28 days), *n* (%)	4 (5%)
Respiratory support duration	
Duration of MV (days), mean ± SD	1.8 ± 5.4
Duration of NIV (days), mean ± SD	29.2 ± 27.0
Duration of O_2_ therapy (days), mean ± SD	19.2 ± 28.5

IQR, interquartile range; MV, mechanical ventilation; NIV, non-invasive ventilation.

## 4 Discussion

This study provides a comprehensive evaluation of a standardized approach for premedicated, video laryngoscopy-assisted LISA in preterm infants less than 32 weeks' gestation. The primary findings indicate a first-attempt success rate of 62% and a relatively high incidence of transient cardiorespiratory instability, particularly desaturations. These transient events did not translate into a high rate of ultimate procedure failure, as 83% of infants successfully avoided mechanical ventilation in the first 72 h of life. These results offer valuable real-world insights into the clinical trade-offs associated with the decision to use premedication for LISA.

The first-attempt success rate of 62% indicates procedural feasibility and aligns with previous studies reporting rates between 52% and 89% (17–22). Evaluation of patient characteristics did not identify statistically significant predictors of first-attempt LISA success in this cohort. In contrast, a significant association was observed with the level of the leading provider, as attendings achieved higher odds of success compared with fellows and interns. These findings suggest that the provider's level of expertise may play a critical role in procedural outcomes and raise the question of which level of training should be recommended to ensure safe performance of LISA while minimizing procedure-related adverse events. Furthermore, the use of video laryngoscopy may increase procedural safety as well as facilitate the training of interns and fellows by enabling other participants to assist with the intervention (23).

The most frequent adverse event was transient desaturation, occurring in 77% of cases. While this proportion is high, it is consistent with the known respiratory depressant effects of fentanyl (15) and with reports from studies using other sedatives and may have been further facilitated by laryngoscopy required for the LISA procedure. A randomized controlled trial of propofol for LISA found desaturations in 91% of sedated infants, while a retrospective study reported 100% using propofol (24, 25). These data indicate that a high incidence of transient desaturation is a common and expected side effect of premedicated LISA. The long term effects of these events are difficult to ascertain, with existing data pointing to no negative impact of short transient desaturations on preterm infants (26). Studies of infants which received LISA without pre-medication showed desaturation rates between 13% and 42% (18, 22, 27, 28), which appears significantly lower. Despite the high frequency of desaturations requiring intervention, only 17% of infants required intubation and mechanical ventilation within 72 h. These findings are comparable to prior studies, with reported rates ranging from 10% to 36.5% when LISA was performed without premedication (3, 19, 20), and from 14% to 42% when premedication was used (24, 25, 29, 30). These results align with recent systematic reviews showing that while analgosedation for LISA is associated with more frequent desaturations and need for PPV during the procedure, it does not significantly affect the overall need for mechanical ventilation within 24–72 h (9, 31). Our findings add to the view that transient cardiorespiratory events can be effectively managed with short-term non-invasive support, in our case 24% of the infants requiring PPV, which is lower in comparison to other studies (20). It is difficult to say to what extent the constellation of desaturations during LISA procedure and reduced pain through the use of analgesics provide long-term effects on the infants. Our approach did not however compromise the primary goal of LISA—avoiding sustained mechanical ventilation. Furthermore, the observed rates of severe IVH (8%), pneumothorax (2%), and early mortality (5%) in our patients cohort are consistent with the favorable safety profile of LISA reported in other studies (18, 20). While some concerns were raised about mortality in preterms of 25–26 weeks' gestation (3) a prospective observational study did not find any association with an increased risk of mortality (32). The conflicting results suggest that the safety and efficacy of LISA may be dependent on the clinical context, patient population and procedural factors such as team experience, or modes of application.

The strengths of this study include the evaluation of a consistently applied, real-world approach and the systematic use of both video laryngoscopy and a defined premedication regimen, reducing operator-dependent variability. However, the retrospective, single-center design with a rather small sample size limits generalizability. The absence of a control group (e.g., LISA without premedication) precludes direct conclusions about the specific effects of fentanyl. A further limitation is the absence of formal pain or comfort assessment; although premedication was intended to reduce pain and distress, this benefit was presumed rather than objectively measured, leaving an important gap for future research.

## 5 Conclusion

In conclusion, this study shows that a standardized procedure for premedicated, video laryngoscopy–assisted LISA is a feasible and effective approach for avoiding mechanical ventilation in most preterm infants under 32 weeks' gestation with RDS. This method was associated with a relatively high rate of transient desaturations, which may nevertheless be an acceptable trade-off for the presumed benefits of analgesia during a painful procedure. Future studies should include prospective, randomized controlled trials comparing different premedication regimens with non-pharmacological comfort measures, incorporating validated pain and comfort scoring, detailed cardiorespiratory monitoring, and long-term neurodevelopmental follow-up, while also focusing on provider characteristics.

## Data Availability

The raw data supporting the conclusions of this article will be made available by the authors, without undue reservation.

## References

[1] Herting E Hartel C Gopel W. Less invasive surfactant administration (LISA): chances and limitations. Arch Dis Child Fetal Neonatal Ed. (2019) 104(6):F655–9. 10.1136/archdischild-2018-316557 31296694 PMC6855838

[2] Schmolzer GM Kumar M Pichler G Aziz K O'Reilly M Cheung PY. Non-invasive versus invasive respiratory support in preterm infants at birth: systematic review and meta-analysis. Br Med J. (2013) 347:f5980. 10.1136/bmj.f5980 24136633 PMC3805496

[3] Dargaville PA Kamlin COF Orsini F Wang X De Paoli AG Kanmaz Kutman HG Effect of minimally invasive surfactant therapy vs sham treatment on death or bronchopulmonary dysplasia in preterm infants with respiratory distress syndrome: the OPTIMIST-A randomized clinical trial. JAMA. (2021) 326(24):2478–87. 10.1001/jama.2021.21892 34902013 PMC8715350

[4] Gopel W Kribs A Ziegler A Laux R Hoehn T Wieg C Avoidance of mechanical ventilation by surfactant treatment of spontaneously breathing preterm infants (AMV): an open-label, randomised, controlled trial. Lancet. (2011) 378(9803):1627–34. 10.1016/S0140-6736(11)60986-0 21963186

[5] Kribs A Roll C Gopel W Wieg C Groneck P Laux R Nonintubated surfactant application vs conventional therapy in extremely preterm infants: a randomized clinical trial. JAMA Pediatr. (2015) 169(8):723–30. 10.1001/jamapediatrics.2015.0504 26053341

[6] Yew R Fleeman M Gowda H. Should premedication be used for less invasive surfactant administration (LISA)? Arch Dis Child. (2023) 108(2):141–3. 10.1136/archdischild-2022-324922 36446482

[7] Reynolds P Bustani P Darby C Fernandez Alvarez JR Fox G Jones S Less-invasive surfactant administration for neonatal respiratory distress syndrome: a consensus guideline. Neonatology. (2021) 118(5):586–92. 10.1159/000518396 34515188

[8] Isayama T Iwami H McDonald S Beyene J. Association of noninvasive ventilation strategies with mortality and bronchopulmonary dysplasia among preterm infants: a systematic review and meta-analysis. JAMA. (2016) 316(6):611–24. 10.1001/jama.2016.10708 27532916

[9] Tribolet S Hennuy N Snyers D Lefebvre C Rigo V. Analgosedation before less-invasive surfactant administration: a systematic review. Neonatology. (2022) 119(2):137–50. 10.1159/000521553 35124678

[10] Boggini T Pozzoli S Schiavolin P Erario R Mosca F Brambilla P Cumulative procedural pain and brain development in very preterm infants: a systematic review of clinical and preclinical studies. Neurosci Biobehav Rev. (2021) 123:320–36. 10.1016/j.neubiorev.2020.12.016 33359095

[11] Durrmeyer X Walter-Nicolet E Chollat C Chabernaud JL Barois J Chary Tardy AC Premedication before laryngoscopy in neonates: evidence-based statement from the French society of neonatology (SFN). Front Pediatr. (2022) 10:1075184. 10.3389/fped.2022.1075184 36683794 PMC9846576

[12] Peterson J den Boer MC Roehr CC. To sedate or not to sedate for less invasive surfactant administration. An ethical approach. Neonatology. (2021) 118(6):639–46. 10.1159/000519283 34628413

[13] McPherson C Haslam M Pineda R Rogers C Neil JJ Inder TE. Brain injury and development in preterm infants exposed to fentanyl. Ann Pharmacother. (2015) 49(12):1291–7. 10.1177/1060028015606732 26369570 PMC4644677

[14] Puia-Dumitrescu M Comstock BA Li S Heagerty PJ Perez KM Law JB Assessment of 2-year neurodevelopmental outcomes in extremely preterm infants receiving opioids and benzodiazepines. JAMA Netw Open. (2021) 4(7):e2115998. 10.1001/jamanetworkopen.2021.15998 34232302 PMC8264640

[15] Murphy CA Goss KC Slater R Ojha S Dargaville PA Gale C. Premedication for less invasive surfactant administration: a narrative review. Arch Dis Child Fetal Neonatal Ed. (2025) 110(3):230–5. 10.1136/archdischild-2024-326947 39389764

[16] Silverman WA Andersen DH. A controlled clinical trial of effects of water mist on obstructive respiratory signs, death rate and necropsy findings among premature infants. Pediatrics. (1956) 17(1):1–10. 13353856

[17] de Kort E Kusters S Niemarkt H van Pul C Reiss I Simons S Quality assessment and response to less invasive surfactant administration (LISA) without sedation. Pediatr Res. (2020) 87(1):125–30. 10.1038/s41390-019-0552-z 31450233 PMC7223491

[18] Gupta V Weinberger B Galanti SG Patel J Kasniya G Kurepa D. Outcomes, safety and health economics of introduction of video laryngoscopy-assisted less invasive surfactant administration. J Perinatol. (2025) 45(4):513–20. 10.1038/s41372-024-02162-4 39578512 PMC12069099

[19] Jahmani T Miller MR da Silva O Bhattacharya S. Application of video laryngoscopy for minimally invasive surfactant therapy: a retrospective comparative cohort study. Biomedicines. (2024) 12(3):618. 10.3390/biomedicines12030618 38540231 PMC10968581

[20] Kurepa D Boyar V Predtechenska O Gupta V Weinberger B Pulju M Video laryngoscopy-assisted less-invasive surfactant administration quality improvement initiative. Arch Dis Child Fetal Neonatal Ed. (2023) 108(6):588–93. 10.1136/archdischild-2023-325357 37028921

[21] Maiwald CA Neuberger P Franz AR Engel C Vochem M Poets CF Clinical evaluation of an application aid for less-invasive surfactant administration (LISA). Arch Dis Child Fetal Neonatal Ed. (2021) 106(2):211–4. 10.1136/archdischild-2020-319792 33023914 PMC7907548

[22] Pichler K Kuehne B Dekker J Stummer S Giordano V Berger A Assessment of comfort during less invasive surfactant administration in very preterm infants: a multicenter study. Neonatology. (2023) 120(4):473–81. 10.1159/000530333 37311430 PMC10614453

[23] Riva T Engelhardt T Basciani R Bonfiglio R Cools E Fuchs A Direct versus video laryngoscopy with standard blades for neonatal and infant tracheal intubation with supplemental oxygen: a multicentre, non-inferiority, randomised controlled trial. Lancet Child Adolesc Health. (2023) 7(2):101–11. 10.1016/S2352-4642(22)00313-3 36436541

[24] Dekker J Lopriore E van Zanten HA Tan R Hooper SB Te Pas AB. Sedation during minimal invasive surfactant therapy: a randomised controlled trial. Arch Dis Child Fetal Neonatal Ed. (2019) 104(4):F378–F83. 10.1136/archdischild-2018-315015 30068669

[25] Descamps CS Chevallier M Ego A Pin I Epiard C Debillon T. Propofol for sedation during less invasive surfactant administration in preterm infants. Arch Dis Child Fetal Neonatal Ed. (2017) 102(5):F465. 10.1136/archdischild-2017-312791 28483817

[26] Poets CF Roberts RS Schmidt B Whyte RK Asztalos EV Bader D Association between intermittent hypoxemia or bradycardia and late death or disability in extremely preterm infants. JAMA. (2015) 314(6):595–603. 10.1001/jama.2015.8841 26262797

[27] Conlon SM Osborne A Bodie J Marasch J Ryan RM Glenn T. Introducing less-invasive surfactant administration into a level IV NICU: a quality improvement initiative. Children (Basel). (2021) 8(7):580. 10.3390/children8070580 34356559 PMC8307302

[28] Klein R Fastnacht L Kribs A Kuehne B Mehler K. LISA eligibility and LISA success in extremely preterm infants: a single-center experience. Neonatology. (2024) 121(4):530–5. 10.1159/000537904 38599191 PMC11318578

[29] Brotelande C Milesi C Combes C Durand S Badr M Cambonie G. Premedication with ketamine or propofol for less invasive surfactant administration (LISA): observational study in the delivery room. Eur J Pediatr. (2021) 180(9):3053–8. 10.1007/s00431-021-04103-1 33954805

[30] Krajewski P Szpecht D Hozejowski R. Premedication practices for less invasive surfactant administration - results from a nationwide cohort study. J Matern Fetal Neonatal Med. (2022) 35(24):4750–4. 10.1080/14767058.2020.1863365 33356691

[31] Moschino L Ramaswamy VV Reiss IKM Baraldi E Roehr CC Simons SHP. Sedation for less invasive surfactant administration in preterm infants: a systematic review and meta-analysis. Pediatr Res. (2023) 93(3):471–91. 10.1038/s41390-022-02121-9 35654833

[32] Hartel C Herting E Humberg A Hanke K Mehler K Keller T Association of administration of surfactant using less invasive methods with outcomes in extremely preterm infants less than 27 weeks of gestation. JAMA Netw Open. (2022) 5(8):e2225810. 10.1001/jamanetworkopen.2022.25810 35943742 PMC9364126

